# Every sector is a health sector: expanding organizational health literacy to advance equity and trust

**DOI:** 10.3389/fpubh.2026.1844399

**Published:** 2026-07-16

**Authors:** Melanie Sampson, Natalie Sampson

**Affiliations:** 1 Literacy Works, Chicago, IL, United States; 2 Department of Health and Human Services, University of Michigan–Dearborn, Dearborn, MI, United States

**Keywords:** health equity, implementation science, organizational health literacy, plain language, social determinants of health

## Abstract

Trust in public institutions is low, while the systems that shape health and wellbeing are often difficult to understand, navigate, and influence. This commentary argues that organizational health literacy (OHL) offers a practical and equity-oriented framework for redesigning those systems, not only in healthcare but across sectors such as education, housing, environmental planning, social services, and across local government. OHL shifts attention from individuals' ability to understand complex information to organizations' responsibility to make information, services, processes, and decisions clear, accessible, culturally responsive, and accountable. Building on public health frameworks that recognize the conditions that produce health, we propose that OHL can help operationalize health equity by reducing administrative burden, improving communication, strengthening navigation support, and creating feedback loops with the people most affected by institutional decisions. We trace the evolution of health literacy from an individual-focused concept to an organizational responsibility, identify theoretical frameworks that can support adoption across diverse sectors, and describe mechanisms such as accreditation, grant requirements, workforce training, equity audits, community accountability structures, and performance measurement. Examples illustrate how unclear communication, fragmented workflows, inaccessible public processes, and nonresponsive documentation can deepen inequities, and how OHL strategies could reduce these harms. Future research should test implementation strategies, measurement approaches, costs, and equity outcomes across sectors.

## Introduction

Trust is essential and necessary for an engaged, democratic, and healthy society, but trust in the U.S. government is at near-historic lows (1–3). Rebuilding trust requires systems that are understandable, transparent, and responsive to the people who use them. Organizational health literacy (OHL) offers a practical framework for improving those systems. OHL refers to the ways in which organizations make it easier for people to find, understand, and use information and services in ways that support their health and overall wellbeing. The concept focuses on the systems, policies, and practices, not just individual skills (4, 5). In practice, OHL emphasizes clear, effective and user-friendly processes through a lens of cultural humility that recognizes and honors the diversity of our communities. It also requires asking the people most affected what they need, what barriers they face, and what solutions would work.

Social, commercial, and political conditions fundamentally shape health outcomes and drive health inequities, making it clear that every sector plays a critical role in public health (6). The vital conditions we need to thrive include sustainable resources, contact with nature, freedom from hazards, nutritious food, safe drinking water, humane housing, safe work with livable wages, opportunities for lifelong learning, and reliable transportation, among others (7). If these conditions are managed through confusing or exclusionary systems, inequities widen, regardless if someone has had a positive experience at their most recent doctor's visit. By implementing OHL, we can more effectively intervene across these diverse settings and sectors, fostering environments that support clear communication and equitable and actionable access to information and services. As public health thinking has evolved, it has increasingly recognized that every sector is a public health sector (8), underscoring the importance of integrating OHL into all facets of society to address and reduce health inequities.

This perspective piece argues that OHL should be applied in and beyond healthcare to advance health equity (9). We discuss how public health approaches and frameworks have evolved to recognize how sectors not traditionally considered health sectors shape health outcomes. We explain why OHL is an appropriate lens to support organizations that govern these sectors, pushing them to be understandable, navigable, responsive, and accountable to the people most affected by their decisions and actions. Finally, we introduce theoretical frameworks and institutional incentives and mechanisms that could support realistic diffusion of OHL across sectors such as education, housing, environmental planning, public agencies, and social services.

## Background: from individual health literacy to organizational responsibility

While the formal concept of OHL only emerged in recent decades, its foundations can be partially traced to earlier civil-rights–era demands for equity and access, such pressures that were later codified in policy and enforced through litigation and regulation. Title VI of the Civil Rights Act of 1964 prohibited discrimination based on race, color, or national origin by federally funded healthcare programs, laying the groundwork for language assistance and equal access, though implementation often fell short of meeting the public's true information needs (10). At the time, most hospitals, clinics, and public health agencies in the United States operated almost entirely in English. There were often no interpreters or translated materials available, and medical staff typically lacked training to communicate effectively with patients who spoke Spanish, Chinese, Polish, or other languages common in immigrant communities. If a patient needed to understand medication instructions, consent forms, diagnosis information, or case management paperwork, they were largely reliant on friends, family, or sometimes strangers to help translate. Beginning in the late 1960s and 1970s, some court cases and federal actions interpreted lack of language access as a type of discrimination, pushing hospitals and agencies to start offering translation services. Later, the Americans with Disabilities Act (ADA) of 1990 required healthcare institutions to provide reasonable accommodations, such as interpreters and accessible materials, to people with disabilities (11). Still, with federal policies in place, many organizations were slow to adapt their systems for diverse populations and, even for those that did, it was clear that improved staff training, increased cultural humility, plain language communication, and other institutional changes were still needed.

In parallel, the concept of health literacy first emerged in the 1970s, with initial efforts focusing on individuals' abilities to read, understand, and use health information. The term is often attributed to Simonds (12), who advocated for new social policies that would incorporate health education and set minimum standards for “health literacy” within K-12 schools. Today, the most cited definition is in a 2000 National Library of Medicine report, which defined health literacy as, “the degree to which individuals have the capacity to obtain, process, and understand basic health information and services needed to make appropriate health decisions” (43). However, research over the following decades revealed that, regardless of literacy level, any individual could be confused by the complexity of health systems, leading to a shift in perspective during the 2000s. At this time, Nutbeam (13) offered a new framework, identifying three levels of health literacy: functional health literacy (i.e., basic reading and understanding of health information), interactive health literacy (i.e., communicating, engaging, and applying information in context), and critical health literacy (i.e., analyzing information, understanding structural determinants, and participating in collective action). Influential reports, such as the Institute of Medicine's “Health Literacy: A Prescription to End Confusion” (44), exposed the limitations of relying solely on individuals to navigate complex health systems, hinting at the need for broader organizational responsibility that would become central to later health literacy conceptualizations. The Plain Writing Act of 2010 also codified the responsibility of federal agencies in communicating in clear, audience-centered communication. It has been effective in building infrastructure and improving some public-facing communications, but also has had weak enforcement and inconsistent implementation, especially for complex materials (14).

In the 2010s, OHL gained traction, with frameworks like the “Ten Attributes of Health Literate Health Care Organizations” providing clear strategies for institutions to make information and services accessible to all (15). Sørenson et al. (16) made the case that health literacy entailed people's knowledge, motivation and competences to access, understand, appraise, and apply health information. Brach (15) proposed strategies related to leadership, workforce training, inclusive design, clear communication, and support through high-risk situations (e.g., care transitions, communications about medicines). These attributes became integrated into policy and practice, notably through the Affordable Care Act and accreditation standards, prompting hospitals, clinics, and universities to improve communication, signage, and patient materials. In 2020, OHL was integrated into the overarching goals of the Department of Health and Human Services' Healthy People framework, with a 2030 goal to: “Eliminate health disparities, achieve improved health for all people, and attain health literacy to improve the health and wellbeing of all” (17). Healthy People 2030 also updates the definition of health literacy to include both individual and organizational components (18).

Today, OHL work largely happens in the healthcare sector, where much progress has been made relative to other fields, and much more progress is needed. Lack of OHL in healthcare costs us as a society. When systems are not set up in accessible, navigable ways, we are more likely to see medical errors, lost productivity, exacerbated health disparities, lawsuits, anxiety, deferred care, mistrust, and preventable morbidity or mortality (45, 46). The current healthcare system entails complex medical jargon, prescription instructions, consent forms, patient education materials, appointment reminders, test results, discharge instructions, portal navigation, and navigation of multiple specialists. Our complex, for-profit U.S. insurance system drives up costs and makes care hard to access and navigate, which contributes to health inequities (19, 20). Claim denials and delays are experienced more frequently by patients with lower incomes and less education, as well as more so by communities of color (21). Research highlights increasing efforts towards OHL, however, such as training healthcare staff in plain language, improving patient education materials, translating vital documents, and redesigning systems for better accessibility (22). Many health systems have implemented policies to assess and improve their own health literacy, and accreditation bodies now increasingly require attention to health literacy domains. However, literature consistently points out persistent gaps. Implementation of OHL varies widely across institutions, with most efforts focusing on written communication and sporadic staff training rather than ongoing, system-wide transformation (15, 47). There are challenges in leadership buy-in, integrating health literacy into workflows, resource allocation, and sustainable collaboration with patients and communities (48). Overall, while healthcare has set important standards and produced a wealth of research and guidance, progress is uneven, and truly embedding health literacy as an organizational value remains a work in progress (49).

Recently, local governments have taken an active role in advancing OHL as well. The Cook County Department of Public Health, with funding from the Centers for Disease Control and Prevention and the U.S. Department of Health and Human Services, launched a toolkit in 2025 to help organizations improve health equity and access through strategies in leadership, workforce development, partnerships, communication, and community engagement (23). Washington, DC also led a city-wide effort with the Advancing Health Literacy Project, funded by the Office of Minority Health, bringing together community partners to create a model supporting non-health organizations in building health literacy and collaborative networks (24). In Virginia, university researchers joined with the Department of Health to assess and strengthen OHL in underserved districts using the Agency for Healthcare Research and Quality toolkit, implementing training workshops and e-newsletters, though ongoing challenges remain in engaging frontline staff and sustaining improvements (25). The University of Maryland School of Public Health Horowitz Center for Health Literacy (26) launched the Maryland Health Literacy Organizational Seal Program for Maryland to recognize institutions that prioritize clear communication.

## Multisectoral OHL as a tool for health equity

For public health professionals, embracing OHL is a natural extension of existing work and core values. Public health has long prioritized strategies like Health-in-All-Policies (27), as well as antiracist frameworks (28) and cultural humility (29), all of which aim to improve equitable access to care and break down silos and systemic barriers. OHL can support these values by offering a practical, actionable framework for institutions to ensure everyone can find, understand, and use health information and services in ways that best serve them and their loved ones.

To address health inequities, we increasingly understand that we must address people and communities holistically to be most effective. People's experiences are shaped by intersecting identities (e.g., race, language, education level, disability status) in ways that can protect health such as strong social support networks, cultural connectedness, access to community-based resources, and self-advocacy and navigation skills. These systems can also compound and deepen health inequities by failing to address biases in the system that impact or harm people (30). For instance, as a form of racial bias, clinicians are less likely to believe or adequately treat pain in Black patients due to false beliefs about biological differences (31, 32). Patients whose primary language is not English may often receive lower-quality communication when professional interpreters are not used consistently (33, 34). In other sectors, for example, schools districts might require families to understand complex disability law to access accommodations, housing agencies might require extensive documentation from people experiencing homelessness, public benefits systems may use confusing eligibility notices, courts may expect self-represented litigants to navigate legal jargon, planning agencies may publish technical environmental notices that exclude residents, or digital-only service systems may exclude people without reliable internet access. OHL has the potential to interrupt biased systems by helping organizations to remove obstacles and create health services that work better for everyone.

If public health can help identify where health is produced, including schools, housing systems, transportation networks, environmental agencies, social services, courts, and public benefit programs, then perhaps OHL can help identify how those institutions can be redesigned so that their health-shaping functions are accessible in practice. OHL focuses on the everyday interfaces between organizations and the public, such as forms, notices, eligibility rules, meetings, referrals, digital portals, interpretation services, complaint processes, staff communication, and feedback loops. This makes OHL especially useful for operationalizing health equity because it translates broad commitments to social determinants, equity and justice into organizational routines that can be implemented, measured, and held accountable.

## Theories, motivations, and mechanisms for OHL uptake

Theoretically, organizations may want to consider theories of organizational change to support OHL uptake. Various theories draw attention to different dimensions of implementation. Diffusion theory highlights how organizations perceive and adopt innovations (35, 36). Future research might explore application of the following: implementation science emphasizes adaptation, context, and process (37, 38). Street-level bureaucracy theory shows how frontline workers shape policy in practice (39). Administrative burden scholarship clarifies how organizational processes can create inequitable costs for users (40). Institutional theory explains how laws, norms, funding requirements, and peer practices influence organizational behavior (41). Because schools, housing agencies, courts, planning departments, public benefits offices, and healthcare organizations operate under different mandates, incentives, staffing structures, and accountability systems, no single theory will likely fully explain how OHL will diffuse or be sustained in every setting. Still, they offer a flexible conceptual toolkit for analyzing barriers, designing context-specific strategies, and evaluating whether OHL efforts become meaningful, sustainable, and equity-oriented rather than symbolic.

Non-health sectors do not need to adopt OHL solely because they affect health. They may adopt OHL because it helps them meet their own institutional goals, such as reducing administrative burden, improving compliance, increasing service uptake, preventing errors, strengthening community trust, and demonstrating accountability. Table 1 highlights ways that OHL may be valuable to nearly any sector.

**Table 1:** Ways OHL may be valuable across sectors.

Values	Potential Benefits of OHL
Equity & accountability	•More equitable access for people who are English language learners, or with disabilities, low digital access, low income, or prior negative experiences with institutions
Trust & legitimacy	•Clearer processes and responsive communication can help rebuild trust in public institutions •Naming and addressing uncertainty transparently
Legal and regulatory compliance	•Language access •Disability access •Civil rights obligations •Due process •Public participation requirements •Plain language requirements •Administrative procedure requirements
Operational efficiency	•Fewer incomplete forms •Fewer missed appointments •Fewer appeals •Fewer repeated calls •Fewer preventable errors •Less staff time spent correcting misunderstandings
Service quality	•Better uptake of services •More appropriate referrals •Improved user experience •Better continuity across agencies, less siloing
Risk management	•Reduced complaints •Reduced litigation risk •Reduced reputational harm •Better documentation of procedural fairness
Performance management	•OHL can align with existing goals in public agencies, schools, housing systems, and social services around access, quality, responsiveness, and equity

In practice, there are many ways this could be accomplished through accreditation and certification, grant requirements, training and workforce development, community accountability structures, standards and guidelines, contract requirements, reporting systems, or equity audits. For instance, equity audits could review whether navigation burdens fall disproportionately on marginalized groups and agencies could track form completion rates, appeal rates, service drop-offs, complaint patterns, language access use, disability accommodation timeliness, and user comprehension, among other metrics. Also, OHL competencies could be built into planning, education, social work, public administration, and law curricula. To begin, OHL should show up in public health curricula and training programs and be encouraged by accrediting bodies, particularly the Council on Education for Public Health (CEPH). For Master of Public Health degrees, CEPH guidelines do indirectly address OHL through competencies related to communication, social determinants of health, and policy and community change (42). CEPH could explicitly name OHL and require it to be taught and assessed so that graduates recognize and act on OHL, not just individual health literacy.

The following composite of hypothetical case studies (box 1) highlight scenarios where an OHL framework may have improved a patient, client, resident, or family's experience towards health equity, with specific OHL principles and strategies named in Table 2. When reviewing these examples, it is worth noting that this is a lot to ask of already overstrained organizations or agencies, but these are steps administrators, clinicians, community health workers, healthcare/school social workers, teachers, or other system navigators can strive towards.

Box 1 **Hypothetical case studies to examine the potential of OHL strategies navigating breast MRI scheduling—patient perspective and system challenges.**

A 42-year-old white woman attended an MRI appointment after an irregular mammogram, following all instructions to arrive prepared and change into a gown. Upon review of paperwork, staff identified that MRI scans were not performed within a specific window of the menstrual cycle, information that had not been previously communicated to the patient by her physician or appointment scheduler.

Although the staff were courteous and apologetic, the patient experienced frustration over the lack of prior notification, resulting in lost time and the inconvenience of having to return for the test. The patient did not have a flexible work schedule and relied on friends and family for rides to medical appointments. She questioned the impact such communication lapses might have for patients with similarly rigid schedules, childcare needs, or limited transportation options.

The administrative process compounded the frustration: subsequent messages about “rescheduling the missed appointment” framed the event as patient error, an implication that persists in her medical record. When calling to reschedule, the patient carefully outlined the menstrual cycle window needed for optimal MRI timing. The scheduler initially offered an unsuitable date, requiring reiteration of her constraints and ultimately referencing the scheduling manager before a satisfactory appointment was found.

**Navigating a 504 plan request—family perspective and system challenges**

A family sought support from their child's public school after noticing that the student's disability was affecting classroom participation, homework, testing, and attendance. The parent had heard that the student might qualify for a Section 504 plan and contacted the school office to ask how to begin. (Note: In the U.S., 504 plans are legally binding documents designed to support students with disabilities in receiving equal access to education by documenting classroom accommodations.)

The school emailed a packet of procedural documents that were lengthy, technical, and available only in English. The parent's primary language was not English, and terms such as “eligibility determination,” “substantial limitation,” and “procedural safeguards” were not explained. The packet did not include translated materials, a checklist, a visual overview, or a clear staff contact to help navigate the process.

Although staff were polite, they instructed the parent to “complete the forms and return the required documentation” before a meeting could be scheduled. The parent was unsure which forms were required, whether medical documentation was necessary, and how long the process should take. Because the parent worked an hourly job with limited flexibility, repeated calls during school hours were difficult.

The administrative process compounded the frustration. Follow-up emails described the request as “incomplete,” implying the delay was due to parent inaction rather than unclear instructions and lack of language access. When the parent called for help, they were transferred between the school office, teacher, counselor, and district 504 coordinator, with each person providing slightly different guidance.

With help from a bilingual community advocate, the family eventually submitted the required information and scheduled a 504 meeting. However, the experience left them questioning how many families, especially whose first language is not English, rigid work schedules, limited digital access, or unfamiliarity with disability procedures—might delay or abandon requests because the system was too difficult to navigate.

**Navigating environmental planning—community perspective and system challenges**

Residents in a historically disinvested neighborhood learned that a permit was being considered for a new industrial facility near their homes, school, and community park. Many were already concerned about asthma, truck traffic, noise, and air quality. Wanting to understand the potential health impact, community members contacted the local planning agency to ask how they could review the proposal and provide input.

The agency directed residents to an online portal with zoning maps, environmental assessments, permit documents, traffic studies, and hearing notices. The materials were lengthy, technical, and written in legal and planning terminology. Terms such as “conditional use permit,” “cumulative impact,” “particulate matter,” and “environmental mitigation” were not explained. Some documents were available only in English, and the portal was difficult to use on a mobile phone.

Although staff were courteous, residents were told they could submit comments or attend a public hearing. The hearing was scheduled during the workday at a government building not easily accessible by public transportation. Residents were unsure which agency had authority, what evidence would be considered, or whether health concerns such as asthma rates and existing pollution burden would influence the decision.

Follow-up notices described the community as having had an “opportunity for public input,” even though many residents could not access or understand the materials in time. With support from an environmental justice organization, residents submitted comments and requested a delay. Still, they questioned how many overburdened communities are expected to repeatedly advocate for their health and safety in systems that are difficult to understand and influence.

**Navigating coordinated entry for housing—client perspective and system challenges**

A parent experiencing homelessness contacted the local coordinated entry system after staying temporarily with friends and then in a shelter with their child. The parent had heard that coordinated entry was the main pathway to housing assistance and called the listed number to begin the process.

The intake process required phone screening, eligibility questions, document submission, and referral to partner agencies. The parent received information from several organizations, but instructions differed about what documents were required, how priority was determined, and how long the process might take. Terms such as “assessment score,” “prioritization list,” “rapid rehousing,” and “program eligibility” were not clearly explained.

Although staff were respectful, the parent was told to check back regularly and keep contact information updated. This was difficult because phone service was inconsistent, transportation was limited, and the parent was managing work, childcare, and shelter rules. The parent was unsure whether their application was complete, whether they were on a waiting list, or whether they needed to contact housing providers separately.

The process became more frustrating when staff at different agencies gave different answers about case status. At one point, the parent was told they had “not followed up,” even though missed communication was partly due to unstable phone access and unclear instructions.

With help from a shelter case manager, the parent completed the required steps and was referred to a housing program. However, the experience raised concerns about how many people may lose housing opportunities because the system is too difficult to navigate.

**Table 2:** OHL principles or strategies to address case study issues.

Shared organizational issue	Breast MRI scheduling case	504 plan access case	Environmental planning	Coordinated entry for housing	OHL principles or strategies to address the issue
Unclear or incomplete communication	The patient was not told in advance that the breast MRI needed to be scheduled within a specific menstrual cycle window. She followed all known instructions but arrived only to learn the appointment could not proceed.	The family received lengthy, technical, English-only procedural documents without clear explanation of what steps were required to request a 504 plan.	Residents were directed to technical zoning, permitting, and environmental documents without plain-language explanations.	Parent received inconsistent information about required documents, eligibility, prioritization, and timelines.	**Use plain language:** Explain technical terms and requirements clearly. **Standardize key communications:** Ensure essential information is provided early and consistently. **Use multiple modalities:** Provide information verbally, in writing, by phone/text/email/portal, and in translated formats.
Lack of navigation support	The patient had to identify the scheduling problem, advocate for the correct timing, and escalate to a scheduling manager to obtain an appropriate appointment.	The parent was transferred among the office, teacher, counselor, and district 504 coordinator, receiving inconsistent guidance and no clear point of contact.	Residents were referred among planning, environmental, zoning, and elected offices.	Parent had to communicate with multiple agencies and was unsure who had current case information.	**Assign navigators or coordinators:** Provide a named contact for complex processes. **Use warm handoffs:** Connect people directly to the right person or office. **Create step-by-step guides:** Include checklists, timelines, and “what happens next” information.
Workflow and process design problems	The scheduling workflow did not appear to include a required prompt about menstrual cycle timing, even though that information was necessary for proper test scheduling.	The 504 request process placed responsibility on the family to interpret complex documents, determine what was missing, and coordinate communication across staff.	Public input process required residents to locate, interpret, and respond to complex documents within limited timeframes.	Coordinated entry required repeated check-ins, document submission, and referrals across agencies.	**Redesign workflows around user needs:** Build critical prompts into scheduling, intake, and review systems. **Reduce administrative burden:** Avoid duplicate steps, repeated explanations, and unnecessary delays. **Use decision-support tools:** Provide staff scripts, checklists, and clear escalation pathways.
Staff knowledge and training gaps	Schedulers or referring providers may not have consistently communicated MRI timing requirements. Staff may not have had clear guidance for handling cycle-dependent scheduling.	School staff were courteous but gave inconsistent information about the 504 process, documentation requirements, and next steps.	Agency staff may have explained only their portion of the permitting process, leaving residents without a full picture.	Staff across agencies gave different answers about case status and requirements.	**Train front-line staff:** Include process requirements, plain-language communication, equity, cultural humility, and trauma-informed practice. **Provide job aids:** Use quick-reference guides for complex or high-stakes processes. **Use teach-back:** Confirm that people understand the next step.
Power imbalance and need for self-advocacy	The patient had to challenge the offered appointment date and explain why it was unsuitable. Patients who are less comfortable advocating may accept an inappropriate appointment or delay care.	The parent had to persist through confusing instructions and multiple transfers. Families who are English language learners or less familiar with school systems may struggle to advocate effectively.	Residents had to organize to influence decisions affecting their air, water, land use, and health.	Parent had to keep following up despite homelessness, unstable phone access, childcare needs, and shelter rules.	**Design systems so people do not have to “fight” for access:** The burden should not fall on individuals to correct system failures. **Normalize questions and concerns:** Create safe channels for clarification and appeal. **Address bias:** Train staff to avoid labeling people as “difficult,” “noncompliant,” or “unresponsive.”
Blaming or inaccurate documentation	Follow-up messages described the situation as a “missed appointment,” implying patient error even though the appointment was not completed because required information had not been communicated.	Follow-up emails described the family's request as “incomplete,” implying parent inaction rather than recognizing unclear instructions and lack of language access.	Notices described residents as having had an “opportunity for public input,” despite inaccessible materials and meetings.	Parent was described as having “not followed up,” despite unstable phone access and unclear instructions.	**Use non-blaming language:** Distinguish between individual action, organizational barriers, and system failures. **Create correction pathways:** Allow patients, families, residents, and clients to challenge inaccurate records or descriptions. **Track system-caused delays:** Use documentation to identify process failures rather than assign blame.
Administrative burden and unequal impact	The patient lost time, had to reschedule, and faced delayed diagnostic testing. The burden would be greater for patients with rigid work schedules, transportation barriers, caregiving responsibilities, or limited health literacy.	The family experienced delays in accessing accommodations. Burden would be greater for families who are English language learners, with limited digital access, hourly jobs, transportation barriers, or unfamiliarity with disability procedures.	Residents had to review technical documents, attend hearings, and organize advocacy while managing work, caregiving, and transportation barriers.	Parent had to maintain contact, gather documents, and follow up while experiencing housing instability.	**Reduce burden:** Simplify forms, minimize repeat contacts, and coordinate internally. **Offer flexible access options:** Provide evening hours, virtual participation, phone support, interpreters, mobile-friendly tools, and transportation-aware scheduling. **Monitor inequities:** Track delays and outcomes by language, race/ethnicity, disability, housing status, transportation access, and other equity indicators.
Weak feedback loops and accountability	It is unclear whether the failed appointment triggered a process review or whether staff had a way to report that patients were not being informed about timing requirements.	It is unclear whether the school tracked why families struggled with 504 paperwork or whether confusing documents were reviewed after delays occurred.	It was unclear whether agencies evaluated whether public input processes were understandable or accessible.	It was unclear whether coordinated entry partners reviewed where clients lost contact or stalled.	**Create feedback loops:** Collect input from users and front-line staff about where processes break down. **Audit outcomes:** Review cancellations, incomplete applications, delayed permits, unresolved housing referrals, and complaints. **Assign accountability:** Identify who is responsible for improving communication, training staff, and revising workflows.
Lack of community or user co-design	MRI instructions may have been designed from a clinical or administrative perspective rather than from the patient's perspective.	504 materials appeared to be designed from a legal or institutional perspective rather than from the family's perspective.	Planning processes centered on technical and legal requirements rather than community understanding and lived experience.	Coordinated entry processes may reflect agency requirements more than the realities of people experiencing homelessness.	**Co-design materials and workflows:** Involve patients, families, residents, clients, advocates, interpreters, and front-line staff. **User-test materials:** Ask intended users to review forms, notices, portals, and instructions before implementation. **Revise based on lived experience:** Treat confusion, delay, and drop-off as signs that the system needs redesign.

## Discussion

Efforts to strengthen OHL face a host of persistent barriers that span education, healthcare, environmental planning, social services, and nearly every sector that interfaces with the public and has implications for public health. Limited funding, lack of leadership commitment, insufficient staff training, and resistance to change all hinder progress. Many organizations grapple with misconceptions about OHL's benefits, entrenched internal biases, and outdated systems that make adaptation difficult. Technological hurdles, such as inaccessible digital platforms, further exclude those most in need. All the while, staff are often overwhelmed by competing priorities and limited time. These challenges are not limited to any one sector but are woven throughout the fabric of public institutions and private entities.

There are important risks to consider, too. OHL could be adopted tokenistically, reduced to checklists or toolkits, or framed as another responsibility for frontline staff without providing the time, authority, technology, or resources needed to implement it. Checklists and toolkits serve as valuable starting points for embedding OHL principles into practice, offering practical guidance and structured frameworks for reflection and improvement. These can and should be integrated into training in public health, policy administration, environmental planning, education, healthcare, and other sectors. Yet their impact is limited if they are not coupled with deeper shifts and commitments. Organizations may also treat plain language as sufficient while ignoring deeper issues of power, eligibility design, surveillance, stigma, and structural inequity.

It is crucial to recognize that many of these barriers and risks do not arise by accident. Many navigation burdens are not accidental. They reflect histories of racial exclusion, ableism, class inequality, language marginalization, and administrative systems designed around institutional convenience rather than public access. OHL interventions therefore cannot be limited to clearer brochures, and they must also examine eligibility rules, documentation requirements, digital-only processes, staff discretion, enforcement practices, and accountability structures that distribute burdens unequally. Efforts to improve OHL must confront not just logistical and technical challenges but deeply embedded social and structural dynamics that require systemic change. Addressing OHL effectively demands confronting these roots, dismantling oppressive practices, addressing harms, and fostering environments where equity, trust, and health literacy are prioritized across every layer of an organization.

Future research should therefore examine how OHL can be measured, which implementation strategies work best in different sectors, and whether OHL interventions reduce administrative burden, improve trust, increase service uptake, and narrow inequities. Comparative case studies, cost-benefit analyses, implementation studies, and evaluations of cross-sector OHL interventions would help determine when, why, and how OHL becomes a durable organizational practice rather than an equity-oriented ideal. Additional questions to pursue: What organizational incentives most strongly predict OHL adoption outside healthcare? Which sectors are most ready for OHL implementation and why? What OHL tools are transferable across sectors, and which require adaptation? How should OHL be measured in schools, housing agencies, planning departments, and social service systems? What governance mechanisms best sustain OHL over time? Does OHL reduce administrative burden? Does OHL improve trust, service uptake, procedural justice, or equity outcomes? How do frontline workers interpret and implement OHL practices? What are the costs and savings associated with OHL implementation?

Extending OHL beyond healthcare is not simply a call for non-health sectors to accept public health responsibility. It is a proposal for making institutions that shape vital conditions more understandable, navigable, responsive, and accountable. Multisectoral health work is not new. What OHL adds is a theoretical and practical lens. For OHL to diffuse beyond healthcare, however, advocates must articulate its value to other sectors, align it with existing incentives, and develop implementation mechanisms that fit real organizational constraints. Future work should test OHL strategies across sectors, measure their effects on administrative burden and equity, and identify governance structures that sustain organizational change.

## Data Availability

The original contributions presented in the study are included in the article/supplementary material, further inquiries can be directed to the corresponding author.
